# Clinicians’ adherence to clinical practice guidelines for cardiac function monitoring during antipsychotic treatment: a retrospective report on 434 patients with severe mental illness

**DOI:** 10.1186/s12888-017-1289-z

**Published:** 2017-03-31

**Authors:** Mirko Manchia, Giorgio Firinu, Bernardo Carpiniello, Federica Pinna

**Affiliations:** 1 Section of Psychiatry, Department of Medical Science and Public Health, University of Cagliari, Via Liguria 13, 09127 Cagliari, Italy; 2 Department of Pharmacology, Dalhousie University, Halifax, NS Canada

**Keywords:** Torsade de point, Electrolytes, ECG, Excess mortality, Side effects

## Abstract

**Background:**

Severe mental illness (SMI) has considerable excess morbidity and mortality, a proportion of which is explained by cardiovascular diseases, caused in part by antipsychotic (AP) induced QT-related arrhythmias and sudden death by Torsade de Point (TdP). The implementation of evidence-based recommendations for cardiac function monitoring might reduce the incidence of these AP-related adverse events. To investigate clinicians’ adherence to cardiac function monitoring before and after starting AP, we performed a retrospective assessment of 434 AP-treated SMI patients longitudinally followed-up for 5 years at an academic community mental health center.

**Methods:**

We classified antipsychotics according to their risk of inducing QT-related arrhythmias and TdP (Center for Research on Therapeutics, University of Arizona). We used univariate tests and multinomial or binary logistic regression model for data analysis.

**Results:**

Univariate and multinomial regression analysis showed that psychiatrists were more likely to perform pre-treatment electrocardiogram (ECG) and electrolyte testing with AP carrying higher cardiovascular risk, but not on the basis of AP pharmacological class. Univariate and binomial regression analysis showed that cardiac function parameters (ECG and electrolyte balance) were more frequently monitored during treatment with second generation AP than with first generation AP.

**Conclusions:**

Our data show the presence of weaknesses in the cardiac function monitoring of AP-treated SMI patients, and might guide future interventions to tackle them.

## Background

Severe mental illness (SMI), including schizophrenia (SCZ), bipolar disorder (BD), major depressive disorder (MDD) and personality disorders (PD), has considerable excess morbidity and mortality [1–3], resulting in a substantially reduced (10-20 years) life expectancy compared to the general population [3, 4]. Indeed, individuals with SMI are susceptible to the development of physical health problems [2]. This high liability appears to be determined by several socioeconomic and clinical [2, 5], treatment-related [6], and genetic [7] factors. For instance, low income, absence of health insurance, and number of concomitant medical conditions seem to explain a large proportion of the excess mortality found in SMI patients [5]. Moreover, a number of severe medical conditions such as nutritional and metabolic diseases, cardiovascular diseases, viral diseases, respiratory tract diseases, musculoskeletal diseases, sexual dysfunction, pregnancy complications, stomatognathic diseases, and possibly obesity-related cancer are more prevalent in SMI patients compared to unaffected individuals [2]. It is possible that these epidemiological findings could be explained, at least in part, by the use of psychotropic treatments, particularly antipsychotics (AP), in SMI patients [6]. Of note, adverse effects on physical health appear to be greatest at higher dosages of AP, when polypharmacy is present, and when vulnerable populations (e.g., old or young) are treated [6]. Finally, individuals with SMI might carry frameworks of genetic susceptibilities that make them more prone to the development of somatic illnesses than the general population (i.e. shared genetic susceptibilities) [7]. These hypotheses are supported by the evidence that higher rates of comorbid somatic illnesses, such as diabetes mellitus type 1 or type 2, were observed in SMI patients in the prepharmacological era [8, 9], and that genetic risk variants for somatic conditions confer also risk for SMI [7, 10].

Another key factor contributing to the excess mortality observed in SMI patients is the presence of obstacles to the implementation of accurate screening and assessment procedures for physical health aspects in SMI patients [11]. It has been proposed that specific strategies, such as screening the patient’s personal and family history at baseline to identify high-risk individuals and to ensure early detection of changes in critical parameters, as well as the adoption of ongoing surveillance methods, might help in setting up adequate standards of care for SMI patients [11]. The implementation of a specific monitoring algorithm is crucial in SMI patients, since the vast majority of them undergo treatment with first and second generation AP, which have specific safety profile issues impacting on cardiovascular, metabolic and endocrine function of treated patients [12]. Concerning cardiovascular function, there is substantial evidence pointing to a relationship between AP treatment and prolongation of QT interval [12, 13]. The frequency of QTc-related arrhythmias in AP-treated psychiatric patients has been estimated at 8%, leading to a rate of sudden unexpected death twice that observed in normal populations and corresponding to 10-15 deaths per 10,000 person-year of observation [14]. Of note, the risk for QTc prolongation and related arrhythmias, such as Torsade de Point (TdP), is substantially increased in the presence of electrolytes imbalance, individual history of syncope, a family history of sudden cardiac death at an early age, congenital QT syndrome, and/or known heart disease [11]. First generation AP, such as haloperidol, thioridazine, sertindole, pimozide, droperidol, as well as second generation AP, such as quetiapine, risperidone, olanzapine, iloperidone, ziprasidone, and amisulpride determine QT prolongation in treated patients [13, 15]. Although the molecular mechanisms linking AP to QT prolongation have not been fully elucidated yet, there is evidence pointing to a direct effect on certain subtypes of myocardial ion channels, particularly the human ether-a-go-go-related gene (HERG) that encodes for a protein associated with a cardiac K^+^ channel involved in regulating repolarizing currents [15–17].

Taken together, this evidence shows that a proportion of the high morbidity and mortality of SMI patients is explained by the presence of cardiovascular conditions, partly caused by the use of AP treatment that can determine QT related arrhythmias and sudden death by TdP. In addition, the lack of appropriate monitoring increases the risk that these events might present as a consequence of, for instance, concomitant electrolytes alterations. The latter parameters are easy to identify, and amenable to treatment, provided that appropriate longitudinal monitoring is set up. In this context, we sought to investigate whether the daily clinical practice routine of psychiatrists is guided by the evidence based recommendations [18–20] on cardiac function monitoring in AP-treated SMI patient and of baseline assessment for the presence of cardiovascular risk factors.

Our primary aim was to investigate the clinicians’ adherence to evidence-based recommendations for cardiac function monitoring, including ECG, biochemical testing (electrolytes), and assessment of individual cardiologic risk factor before and after starting AP treatment in real life clinical setting, as well as for the prescription of psychotropic drugs at low risk for QT prolongation when indicated. The secondary aims of this study were to assess whether the presence of risk factors for QT-related arrhythmias and TdP, such as cardiac or vascular comorbidities, in AP-treated patients impacted on 1) the choice of AP, or other psychotropic treatment such as antidepressants or mood stabilizers, with low risk for QT prolongation, and 2) on the choice of a specific AP pharmacological class (i.e. first generation versus second generation AP). To this end, we performed a retrospective assessment of 434 AP-treated SMI patients longitudinally followed-up at an academic community mental health center.

## Methods

### Patient sample

We conducted a retrospective assessment of 434 SMI patients followed up longitudinally at the Section of Psychiatry of the Department of Medical Science and Public Health, University of Cagliari, Cagliari, Italy. All patients gave written and verbal consent to allow reanalysis of clinical data for research purposes. We collected detailed clinical data for the reference period starting on 1 January 2010 and ending on 30 April 2015. Patients were included in the study if: 1) they had a diagnosis of psychotic disorder, mood disorder, or PD according to Diagnostic Statistical Manual (DSM)-IV-TR criteria [21], and 2) treatment with AP (either first or second generation) was started during the reference period. No exclusion criteria were applied.

### Data extraction protocol

Longitudinal data on the AP treatment used during the reference period, both as monotherapy and combinatorial AP treatment, were extracted for each patient. As a substantial proportion of the sample changed AP therapy over the reference period, multiple longitudinal treatment data were present for each patient. We also extracted data concerning the combinatorial treatment of AP with other psychotropic drugs that increased the risk of QT-related arrhythmias and TdP.

**Fig. 1:**
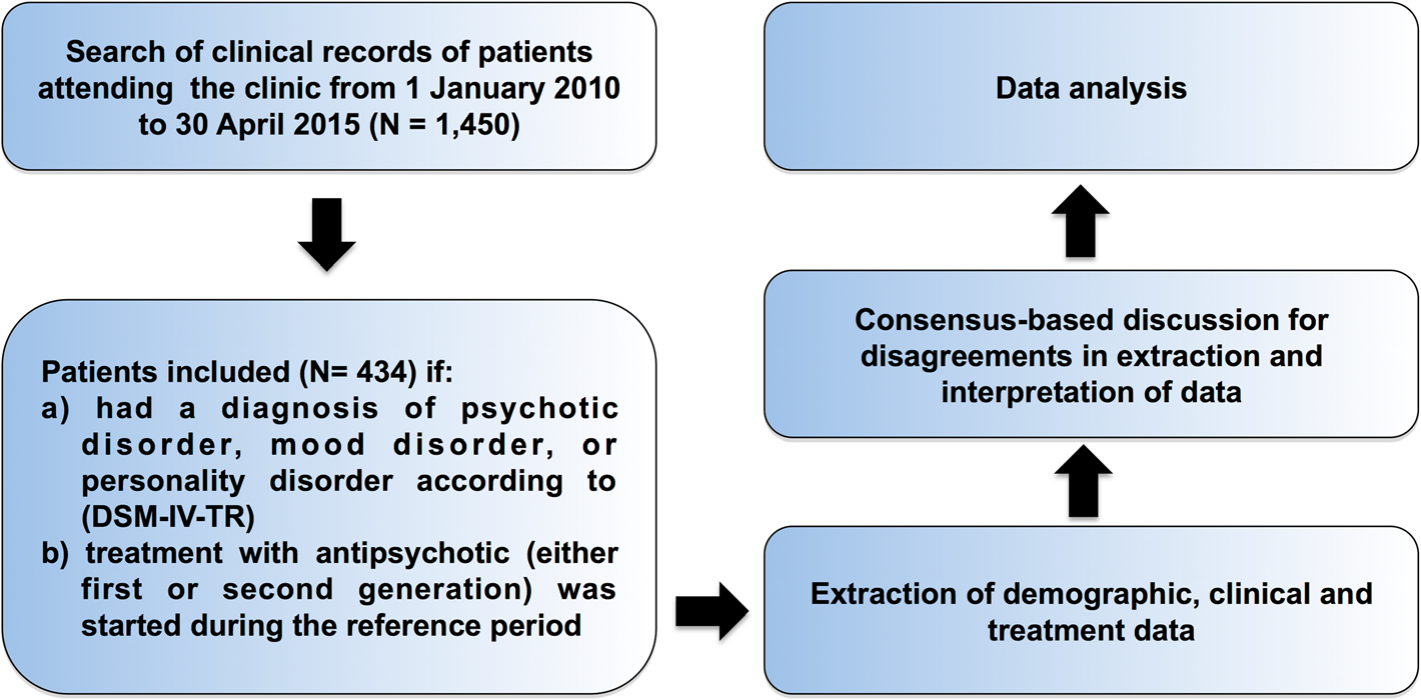
Flowchart illustrating the selection process of clinical records and data analysis. DSM-IV-TR: Diagnostic Statistical Manual-IV-Text Revision

We performed a systematic chart review of the patients observed at our community health center during the reference period. A description of the selection process of clinical records and data analysis is detailed in Fig. 1. The following demographic, clinical and treatment variables were collected and coded as dummy variables whenever appropriate: 1) gender, 2) date of birth, 3) age, 4) marital status, 5) education, 6) employment, 7) age class (< 20 years, between 21 and 30 years, between 31 and 40, > 40 years), 8) age at onset, 9) main psychiatric diagnosis (psychotic disorder, mood disorder, PD), 10) secondary psychiatric diagnosis according to DSM-IV-TR [21] (anxiety disorder, PD, mood disorders, substance abuse or dependence disorder, somatoform disorders, eating disorders, other psychiatric disorders such as dementia, mental retardation, impulse control disorders, autism), 11) presence/absence of comorbid somatic illnesses as described in Table 1, 12) illness duration (dichotomized on the basis of the median value of illness duration after assessing the distributional properties of illness duration: ≤ 3 years or >3 years), 13) AP treatment (active agent), 14) AP risk category for TdP, 15) pharmacological class of AP (first- versus second-generation AP), 16) presence/absence of baseline ECG testing (before AP treatment), 17) presence/absence of personal history of pre-existing cardiac disease, including heart failure, myocardial infarction, cardiac arrhythmias, and myocarditis, 18) presence/absence of positive familial history of cardiac disease, 19) lifetime assumption of pharmacological treatments determining QT prolongation, 20) use of pharmacological treatments determining QT prolongation during AP therapy, 21) presence/absence of electrolytes levels before AP treatment and frequency of testing, 22) presence/absence of electrolytes levels during AP treatment and frequency of testing, 23) presence/absence of ECG testing during AP treatment and frequency of testing. Furthermore, we created the new categorical variable “Number of cardiac function parameters tested” from the sum of the following dummy variables: presence/absence of baseline ECG testing (before AP treatment), presence/absence of ECG testing during AP treatment, presence/absence of electrolytes levels before AP treatment, presence/absence of electrolytes levels during AP treatment, and presence/absence of personal history of pre-existing cardiac disease, including heart failure, myocardial infarction, cardiac arrhythmias, and myocarditis.

**Table 1:** List of comorbid somatic disorders identified in our systematic chart review

Comorbid somatic illness	
Cardiac comorbidities	Congenital and acquired heart conditions
	Arrhythmias
	Previous myocardial infarctions
	Valvular hearth diseases
	Coronary artery diseases
Vascular comorbidities	Arterial hypertension
	Atherosclerotic cardiovascular disease
	Cerebrovascular diseases
	Aneurisms
	Chronic venous insufficiency
	Arterial stenosis
Hematological comorbidities	Monoclonal gammopathy
	Lymphomas
	Myelodysplastic syndromes
Endocrine comorbidities	Hyper/hypothyroidism
	Hashimoto’s thyroiditis
	Goiter
	Pituitary adenomas
Gastrointestinal comorbidities	Chronic inflammatory bowel diseases
	Gastroesophageal reflux disease
	Peptic ulcer disease
	Hiatus hernia
	Celiac disease
Infectious disease comorbidities	Hepatitis B virus
	Hepatitis C virus
	Human immunodeficiency virus
Metabolic comorbidities	Carbohydrate intolerance
	Insulin resistance
	Diabetes mellitus type I and II
	Hypercholesterolemia
	Hyperlipidemia
	Pancreatitis
	Hyperuricemia
	Liver diseases
Kidney conditions	Kidney failure
	Proteinuria
	Hematuria
Neurological comorbidities	Multiple sclerosis
	Epilepsy
	Brain atrophy
	Autonomic disturbances in the limbs
	Arnold-Chiari syndrome
	Parkinson’s disease
Rheumatic diseases	Rheumatoid arthritis and seronegative arthritis,
	Fibromyalgia
	Osteoporosis
	Sjögren syndrome
	Systemic lupus erythematosus
Miscellaneous comorbid diseases	Respiratory problems
	Alterations of hemostasis
	Skin diseases
	Allergies
	Diseases of the musculoskeletal system
	Cancer and organ hyperplasia
	Chromosomal syndromes
	Nephrolithiasis

Data were extracted by one author (G.F.) and subsequently verified with senior investigators (F.P., B.C.). Disagreements in extraction and interpretation of data were resolved with consensus-based discussion.

### Torsade de point risk category of psychotropic drugs

We classified AP according to their risk of inducing QT-related arrhythmias and TdP using the database developed and maintained at the Center for Research on Therapeutics University of Arizona (last access: 2 July 2015) [19]. Briefly, AP and other psychotropic treatments were categorized in 3 classes of risk: A) drugs with known risk of inducing TdP, B) drugs with possible risk of inducing TdP, and C) drugs with conditional risk of TdP. Drugs in class A have substantial evidence supporting their role in determining QT interval prolongation and are clearly associated with a risk of TdP, even when taken as directed in official labeling. Drugs in class B have substantial evidence supporting their role in determining QT interval prolongation but there is insufficient evidence at this time that these drugs, when used as directed in official labeling, are associated with a risk of causing TdP. Finally, for drugs in class C there is substantial evidence supports their association with a risk of TdP but only under certain conditions (e.g. excessive dose, hypokalemia, congenital long QT or by causing a drug-drug interaction that results in excessive QT interval prolongation) [19].

### Statistical analysis

Our null hypotheses were: 1) no association between the cardiac function monitoring parameters (ECG before AP treatment, ECG during AP treatment, electrolytes levels before AP treatment, electrolytes levels after AP treatment association, personal history of pre-existing cardiac disease) both as single independent dummy variables and as total number of parameters, and the TdP risk category; 2) no association between the cardiac function monitoring parameters (ECG before AP treatment, ECG during AP treatment, electrolytes levels before AP treatment, electrolytes levels after AP treatment association, personal history of pre-existing cardiac disease) both as single independent dummy variables and as total number of parameters, and the AP pharmacological class; 3) no association between the presence of risk factors for QT-related arrhythmias and TdP, such as cardiac or vascular comorbidities, and the choice of AP, or other psychotropic treatment such as antidepressants or mood stabilizers, with low risk for QT prolongation, or the choice of a specific AP pharmacological class. To this end, we studied continuous and categorical clinical variables using univariate analysis (*t* test or contingency tables as appropriate). When one or more cells had expected values of 5 or less Fisher’s exact test was used in 2 × 2 contingency tables and bootstrap with 1000 samples in larger tables. Non-parametric tests were used when data violated the assumption of normality. We analysed the distributional properties of continuos data assessing normality with the Kolmogorov-Smirnov test. The variables that showed a significant association with the outcome of interest (TdP risk category or AP pharmacological class) were subsequently included into a multinomial or binary logistic regression model, as appropriate, to correct for age and gender. Specifically, binary logistic regression was used when the dependent variable had two possible discrete outcomes. Instead, we used multinomial logistic regression analysis in the case of a dependent variable with more than two possible discrete outcomes, such as the TdP category. Statistical significance was set at *α* = 0.05. Our sample had more than 90% of statistical power to detect an effect size *w* = 0.5 considering an *α* = 0.05 and degrees of freedom (df) = 6. All statistical analyses were carried out with IBM® SPSS® Statistics version 22.0.0.0 (64 bit), with the exception of power analysis which was performed with G*Power (Version 3.1.9.2).

## Results

### Sample characteristics

The sample was comprised of 434 SMI patients with a mean age of 49.1 ± 14.7 years. The mean age of onset was 30.1 ± 16.1 years with 218 (50.2%) women. The majority of patients (85.5%) had illness duration longer than 3 years. One hundred eighty-eight (43.3%) patients were diagnosed with psychotic disorder, 231 (53.2%) had a mood disorder, and 15 (3.5%) had a PD. One hundred forty-eight (34.1%) patients had a secondary psychiatric diagnosis. Concerning the pattern of comorbidities, 217 (50.0%) had comorbid somatic illnesses, 20 (4.6%) had cardiac conditions, and 49 (11.3%) had vascular diseases. The sample characteristics are detailed in Table 2.

**Table 2:** Characteristics of the study sample (*N* = 434)

Variable	
Women, N (%)	218 (50.2)
Age (mean ± SD)	49.1 ± 14.7
Age of onset (mean ± SD)	30.1 ± 16.1
Age class, N (%)	
≤ 20 years	0 (0.0)
21-30 years	42 (9.7)
31-40 years	91 (21.0)
> 40 years	301 (69.4)
Illness duration >3 years, N (%)	371 (85.5)
Marital status, N (%)	
Single	237 (54.6)
Married/Cohabiting	126 (29.0)
Divorced	40 (9.2)
Widowed	20 (4.6)
NA	11 (2.5)
Employment, N (%)	
Employed	106 (24.4)
Student	28 (6.5)
Retired	79 (18.2)
Unemployed	184 (42.4)
NA	37 (8.5)
Main psychiatric diagnosis, N (%)	
Psychotic disorder	188 (43.3)
Mood disorder	231 (53.2)
Personality disorder	15 (3.5)
Presence of secondary psychiatric diagnosis, N (%)	148 (34.1)
Presence of comorbid somatic illnesses, N (%)	217 (50.0)
Presence of cardiac comorbidities^a^, N (%)	20 (4.6)
Presence of vascular comorbidities, N (%)	49 (11.3)

*SD* standard deviation

*NA* not available

^a^2 missing datas

### Association between cardiac function parameters and TdP risk category

A total of 1348 longitudinal data points on AP treatments were extracted. Each patient received on average 3.1 ± 4.2 AP treatments over the reference period. We first examined each cardiac function parameter with respect to the TdP risk category. Electrolyte and ECG testing baseline were significantly more frequent for AP with known risk of inducing TdP (Category A) and for AP with drugs with possible risk of inducing TdP (Category B) compared to the Category C (both *p* = 0.002, Table 3). These associations remained significant when we corrected for age and gender in the multinomial logistic analysis, with higher rates of baseline electrolytes testing in Category A AP (*p* = 0.006, OR = 0.1) and Category B (*p* = 0.02) compared to category C, as well as a higher rate of baseline ECG testing in Category A AP (*p* = 0.008, OR = 0.19). The number of cardiac function parameters tested was not significantly associated with the TdP risk category (*p* = 0.41, Table 3).

**Table 3:** Association between cardiac function parameters and Torsades de Point (TdP) risk category of antipsychotics

	AP Torsades de Point risk category, *N* (%)				
Monitoring parameters	A	B	C	*χ* ^*2*^	*P-value*
Presence of ECG testing before AP treatment	48 (29.6)	229 (20.0)	3 (1.1)	12.9	0.002
Presence of ECG testing during AP treatment	46 (28.4)	375 (32.8)	16 (38.1)	1.9	0.3
Presence of electrolytes levels before AP treatment	47 (29.0)	246 (21.5)	2 (4.8)	12.1	0.002
Presence of electrolytes levels during AP treatment	51 (31.5)	358 (31.3)	18 (42.9)	2.5	0.3
Presence of personal history of pre-existing cardiac disease	5 (3.1)	57 (5.0)	4 (9.5)	3.1	0.2
Number of cardiac function parameters tested					
0	63 (38.9)	519 (45.4)	20 (47.6)	10.3^a^	0.41^a^
1	27 (16.7)	190 (16.6)	5 (11.9)		
2	55 (34.0)	298 (26.0)	13 (31.0)		
3	8 (4.9)	73 (6.4)	4 (9.5)		
4	9 (5.6)	56 (4.9)	0 (0.0)		
5	0 (0.0)	8 (0.0)	0 (0.0)		

Category A: drugs with known risk of inducing TdP

Category B: drugs with possible risk of inducing TdP

Category C: drugs with conditional risk of TdP

*AP* antipsychotic

*ECG* electrocardiogram

^a^ *χ* ^*2*^1000 sample bootstrap

### Association between cardiac function parameters and AP pharmacological class

The analysis of each cardiac function parameter with respect to the AP pharmacological class showed a higher rate of ECG and electrolyte testing during treatment with second generation AP (*p* = 0.01 and *p* = 0.02, respectively) (Table 4). These associations remained significant in the logistic regression model correcting for age and gender (*p* = 0.008, OR = 1.4 and *p* = 0.014, OR = 1.4, respectively). We also found a trend for association between the number of cardiac function parameters tested and the pharmacological class of AP (Table 4). Specifically, the higher the number of parameters tested (from 3 to 5), the higher the frequency of testing in second generation compared to first generation AP.

**Table 4:** Association between cardiac function parameters and pharmacological class of antipsychotics

	Pharmacological class of antipsychotics			
Monitoring parameters	First generation	Second generation	*χ* ^*2*^	*P-value*
Presence of ECG testing before AP treatment	80 (21.5)	200 (20.5)	0.2	0.7
Presence of ECG testing during AP treatment	101 (27.2)	336 (34.4)	6.5	0.01
Presence of electrolytes levels before AP treatment	81 (21.8)	214 (21.9)	0.004	1
Presence of electrolytes levels during AP treatment	100 (26.9)	327 (33.5)	5.5	0.02
Presence of personal history of pre-existing cardiac disease	26 (7.0)	40 (4.1)	4.8	0.03
Number of cardiac function parameters tested				
0	165 (44.4)	437 (44.8)	11.4^a^	0.04^a^
1	70 (18.8)	152 (15.6)		
2	108 (29.0)	258 (26.4)		
3	14 (3.8)	71 (7.3)		
4	15 (4.0)	50 (5.1)		
5	0 (0.0)	8 (0.8)		

*AP* antipsychotic

*ECG* electrocardiogram

^a^ *χ* ^*2*^1000 sample bootstrap

### Impact of the presence of risk factors for QT-related arrhythmias and TdP on the choice of AP in terms of TdP risk category or pharmacological class

We found that the choice of AP Category C (i.e. drugs with conditional risk of TdP), was significantly overrepresented compared to the other two categories when individual risk factors for QT-related arrhythmias, such as comorbid somatic illnesses, cardiac comorbidities and vascular comorbidities were present (Table 5). These associations remained significant when correcting for age and gender, with a higher rate of use of TdP risk category C AP when cardiac comorbidities (*p* = 0.04, OR = 0.2), and vascular comorbidities (*p* = 0.004, OR = 0.2) were present. Use of pharmacological treatments determining QT prolongation during AP therapy was significantly underrepresented in TdP risk category A and B AP compared to the category C (*p* = 0.003), a finding that remained significant when correcting for age and gender (*p* = 0.039, OR = 8.45 for TdP Category A and *p* = 0.015, OR = 11.7 for TdP Category B). No association of individual risk factors for the development for QT-related arrhythmias and TdP was found with the AP pharmacological class (Table 6). Use of pharmacological treatments determining QT prolongation during AP therapy was significantly underrepresented in first generation AP compared to second generation AP (*p* = 0.001, Table 6). This finding remained significant when correcting for age and gender in the binary logistic regression model (*p* = 0.001, OR = 0.6).

**Table 5:** Association between individual risk factors for QT-related arrhythmias and Torsades de Pointes (TdP), and TdP risk category of antipsychotics

	AP Torsades de Point risk category, *n* (%)				
Factor	A	B	C	*χ* ^*2*^	*P-value*
Presence of comorbid somatic illnesses	82 (50.6)	556 (48.6)	29 (69.0)	6.9	0.03
Presence of cardiac comorbidities	2 (1.2)	45 (3.9)	4 (9.5)	6.8^a^	0.03
Presence of vascular comorbidities	10 (6.2)	123 (10.8)	11 (26.2)	14.0	0.01
Use of pharmacological treatments determining QT prolongation during AP therapy	134 (82.7)	885 (77.4)	41 (97.6)	11.6	0.003

Category A: drugs with known risk of inducing TdP

Category B: drugs with possible risk of inducing TdP

Category C: drugs with conditional risk of TdP

*AP* antipsychotic

^a^ *χ* ^*2*^1000 sample bootstrap

**Table 6:** Association between individual risk factors for QT-related arrhythmias and TdP, and pharmacological class of antipsychotics

	Pharmacological class of antipsychotics			
Factor	First generation	Second generation	*χ* ^*2*^	*P-value*
Presence of comorbid somatic illnesses	191 (51.3)	476 (48.8)	0.7	0.4
Presence of cardiac comorbidities	17 (4.6)	34 (3.5)	0.9	0.35
Presence of vascular comorbidities	37 (9.9)	107 (11.0)	0.3	0.6
Use of pharmacological treatments determining QT prolongation during AP therapy	316 (84.9)	744 (76.3)	11.9	0.001

*AP* antipsychotic

*ECG* electrocardiogram

## Discussion

The excess morbidity and mortality observed in SMI patients is partly determined by clinical, health system-related, and treatment factors that are modifiable, provided that adequate measures for longitudinal monitoring are undertaken. In our retrospective assessment of 434 SMI patients, we found that psychiatrists performed ECG and electrolyte testing before starting AP treatment on the basis of the risk carried by AP of determining QT prolongation and TdP, but not on the basis of AP pharmacological class. However, second generation AP were more frequently monitored than first generation AP, in terms both of ECG and electrolyte balance, during the course of treatment. Of interest, baseline assessment of risk factors for QT-related arrhythmias and TdP had an effect on the choice of AP treatment leading to the use of drugs with a relatively low TdP risk. This resulted also in a specific prescribing pattern of concomitant treatments increasing the likelihood of QT prolongation during AP therapy, which was higher for Category C AP compared to the other two categories.

Several of these results deserve a comment. Our finding that baseline ECG and electrolyte testing was more frequent with AP at higher risk for QT-related arrhythmias and TdP is consistent with evidence based recommendations that baseline testing of SMI patients can reduce the risk of cardiac events over the course of AP treatment [11, 22]. However, longitudinal cardiac function monitoring was not regularly performed over the course of the reference period for any of the three TdP risk category AP. Several reasons might explain this observation. First, the negative symptomatological core of SMI, particularly SCZ and BD, might determine lack of drive and decreased energy levels that can reduce help-seeking and physical health check-ups [23]. Second, it is plausible that the lack of insight into their mental and physical health condition, as well as the presence of thought disorder such as delusion of control and paranoid/persecutory delusions, can explain the resistance of SMI patients to perform longitudinal periodic testing. Finally, the cognitive decline observed in SMI patient might also influence the lack of adherence to cardiac function monitoring. On the other hand, ECG and electrolyte testing during the reference period were more frequently performed in second generation than in first generation AP. This observation might derive from two factors. First, psychiatrists might be more aware of the effects of second generation AP on QT-related prolongation compared to first generation AP given that the former were more recently introduced into the market. Moreover, second generation AP might have higher rate of prescription given the more favorable side effect profile compared to first generation AP [24].

Another interesting finding was that psychiatrists of our community health center prescribed AP at low risk of inducing TdP (Category C) when individual risk factors for QT-related arrhythmias were present. This result is consistent with evidence-based recommendations indicating that baseline screening for cardiovascular risk factors should be performed in order to facilitate targeted treatment-decision making [11]. Of importance, we also identified specific prescribing patterns where AP at low risk of inducing TdP (Category C) resulted more frequently associated with concomitant treatments at increased risk of QT-related arrhythmias. This finding is of particular importance given that TdP might result from additive effect of drugs on cardiac ion channels and might also derive from pharmacokinetic interactions increasing the bioavailability of the interacting drugs [14].

Our results should be interpreted in the context of some limitations. First, the retrospective nature of our study did not permit to assess the temporal relationship between cardiac function testing and the beginning of AP treatment. However, our data were based on accurate and detailed longitudinally collected clinical information. Second, our sample population attended a community mental health center and consequently our results might lack generalizability and be susceptible to selection bias. Third, we could not test for the effect of psychiatry training on clinical monitoring as well as treatment decision-making. It is conceivable that more senior psychiatrists could have higher awareness of the need of performing ECG and biochemical testing in AP treated SMI patients, as well as be more knowledgeable about the side effect profiles of the psychotropic treatment. Interestingly, a survey of junior psychiatrists’ knowledge of ECG monitoring appears to support the latter hypothesis [25]. Fourth, our sample size, although not negligible, might have not been adequately powered to identify pattern of associations with small to moderate magnitude of effect size as shown by our power analysis. Fifth, our findings might have been affected by the lack of stability in longitudinal clinical assessments and data collection. Finally, we limited our reference period to five years. An extension of the reference period for the retrospective assessment, as well as the use of a prospective design, could lead to the identification of some of the obstacles hindering the implementation of evidence-based recommendations for cardiac function monitoring in AP-treated SMI patients.

## Conclusions

Our retrospective study led to the identification of weaknesses in the adherence to cardiac function monitoring guidelines in SMI patients treated with AP in real life clinical practice. Our results appear concordant with previous research showing that clinicians tend to perform poorly at monitoring cardiovascular health in this patient population [26–28]. Indeed, Roberts et al. found that SCZ patients were half as likely as asthma controls to have blood pressure and cholesterol levels recorded [26]. Patients with SMI over 60 years of age were lees likely to receive annual cardiovascular screening than the general population despite higher risk of developing cardiovascular diseases [27]. And cardiovascular monitoring appeared to be poor even in SCZ patients treated with clozapine, which is associated with potentially life-threatening cardiac conditions [28].

In conclusion, our study highlights the need for the development of specific educational programs to facilitate the application of adequate cardiovascular monitoring in clinical setting. These programs should focus on increasing the knowledge on the safety profile of AP with the aim of developing TdP class-specific monitoring guidelines. A more pragmatic approach toward cardiovascular function monitoring of SMI patients should also be based on a closer interaction with general practitioners. Should these health policies be implemented thoroughly, the care, and the quality of life, of SMI patients will improve substantially.
